# Hyperglycemia in the diabetic range, but not previous diagnosis of diabetes mellitus, is an independent indicator of poor outcome in patients hospitalized for severe COVID-19

**DOI:** 10.1007/s00592-025-02507-1

**Published:** 2025-05-02

**Authors:** Alessandra Dei Cas, Raffaella Aldigeri, Elisa Eletto, Andrea Ticinesi, Antonio Nouvenne, Beatrice Prati, Angela Vazzana, Monica Antonini, Valentina Moretti, Emanuela Balestreri, Valentina Spigoni, Federica Fantuzzi, Silvia Schirò, Livia Ruffini, Nicola Sverzellati, Tiziana Meschi, Riccardo Bonadonna

**Affiliations:** 1 Endocrinology and Metabolic Diseases, Azienda Ospedaliero-Universitaria of Parma, Via Gramsci 14, 43126 Parma, Italy; 2 Department of Medicine and Surgery, University of Parma, Parma, Italy; 3 Department of Care Continuity and Multicomplexity, Azienda Ospedaliero-Universitaria of Parma, Parma, Italy; 4 Nuclear Medicine, Azienda Ospedaliero-Universitaria of Parma, Parma, Italy; 5 Radiological Sciences, Azienda Ospedaliero-Universitaria of Parma, Parma, Italy; 6 Endocrinology, Diabetology and Metabolic Diseases, University of Verona and University Hospital of Verona, Verona, Italy

**Keywords:** COVID-19, Diabetes mellitus, Hyperglycemia, Mortality

## Abstract

**Aims:**

Diabetes mellitus (DM) and hyperglycemia are associated with poor outcome(s) in COVID-19 hospitalized patients, but their independent impact on prognosis remains unclear. We aimed to assess the impact of DM and hyperglycemia on COVID-19 outcomes.

**Methods:**

Clinical data/records from COVID-19 patients admitted to the Parma University-Hospital (February 23rd to March 31st, 2020) were retrieved and analysed (NCT04550403). Fasting plasma glucose (FPG), inflammatory markers and the main biochemical variables were collected at admission. Patients underwent chest high-resolution CT and arterial blood gas analysis to determine the PaO_2_/FiO_2_ ratio (P/F ratio). The primary outcome was a composite of intensive care unit admission and/or death.

**Results:**

Among 756 subjects, 143 (19%) had DM. These patients were older with higher comorbidity rates. The primary outcome occurred in 61.5% DM patients versus 43.4% without DM (*p* < 0.001). In multivariable analysis (accuracy UC = 0.93), older age, cardiovascular and kidney diseases, FPG ≥ 126 mg/dl, C-reactive protein, and P/F ratio, but not previous DM, were independent risk indicators.

**Conclusions:**

DM indicated poor COVID-19 outcomes, but not when adjusted for other clinical variables/comorbities, suggesting that its impact was mostly driven by concomitant factors. The independent role of fasting hyperglycemia points to the need for further research on its contribution to COVID-19.

**Supplementary Information:**

The online version contains supplementary material available at 10.1007/s00592-025-02507-1.

## Introduction

On 11 March 2020, the World Health Organization (WHO) declared the coronavirus (COVID-19) outbreak, caused by the severe acute respiratory syndrome coronavirus 2 (SARS-CoV-2), a global pandemic [1]. Northern Italy, which accounted for over 70% of the Italian’s cases, was the first Western country hit by COVID-19 outbreak with the first case reported on January 30^th^. In 2020 in the Emilia Romagna Region, a 20.2% excess in mortality compared to historical trends, both in men and women after adjusting for age [2], was shown. This excess mortality from COVID-19 was significantly higher in individuals with comorbidities such as hypertension, obesity and diabetes mellitus (DM) [3, 4].

In Italy, over one third of deaths associated with COVID-19 occurred in individuals with DM [5] which, according to EpiCentro– Istituto Superiore della Sanità (ISS) data, was the second most frequent reported comorbidity (29.3%) in patients who died from COVID-19 [6]. These data on the impact of DM on severe COVID-19 outcomes have been also confirmed by worldwide evidence [5, 7–11].

Several DM-associated mechanisms may account for a worse prognosis in COVID-19 patients. Chronic inflammation in DM may exacerbate the inflammatory storm due to the host response to COVID-19 infection [12]. In addition, DM itself or its associated treatments, such as angiotensin-converting enzyme (ACE) inhibitors, may influence the severity of COVID-19 by over-expressing the ACE2 receptor, a key entry point for the virus into cells​ [4]. Ultimately, subjects with DM also exhibit a higher overall risk of infections, particularly lower respiratory tract infections of viral etiology, due to multiple immune system perturbations [13].

Of note, hyperglycemia at admission, independently of DM status, has also been reported to be a strong risk factor for poor outcome(s) in patients hospitalized for COVID-19 [11, 14, 15]. Hyperglycemia may amplify the state of over-inflammation and immune dysregulation [16], as it can trigger the cytokine storm by favoring pro-inflammatory macrophage polarization, dysregulating T cells responses, inducing oxidative stress and impairing antigen presentation in dendritic cells (DCs) [16, 17]. In addition, it may facilitate SARS-CoV-2 infection and replication by inducing aberrant glycosylation of the ACE2 receptor, enhancing the binding of SARS-CoV-2 [16, 18, 19].

This study aims to assess the impact of DM and/or hyperglycemia on adverse COVID-19 outcomes [intensive care unit (ICU) admission and/or death] in a large group of patients hospitalized for severe forms of COVID-19 in a large hub in Northern Italy during the first pandemic wave.

## Methods

### Study population

This is an observational, single-centre, retrospective cohort study involving consecutive patients admitted during the first SARS-COV-2 pandemic wave (23^rd^ February 2020-31^st^ March 2020) and managed exclusively in the Covid-1 macro-unit of the University Hospital of Parma (Italy) (NCT04550403). The study was conducted in accordance with the principles of the Declaration of Helsinki and received approval by the local ethic committee (Comitato Etico AVEN, Italy, 15^th^ July 2020). Informed consent was obtained from all contactable living subjects. The study included individuals with a positive nasal-pharyngeal swab for SARS-CoV-2 (real-time PCR) and/or a chest high-resolution computed tomography (HRCT) scan with typical signs of COVID-19 interstitial pneumonia. Patients who were immediately transferred to other departments or sanitary residence care upon admission were excluded from this analysis due to the inability to determine the primary endpoint.

### Data collection

A detailed description of data collection is reported in Supplementary Information. Data were retrieved from inpatient medical records including *(i)* demographic data (age and gender), *(ii)* past medical history [hypertension, atrial fibrillation (AF), renal (chronic kidney disease, CKD) or heart (congestive heart failure, CHF) failure, cancer, chronic obstructive pulmonary disease (COPD), cardiovascular event(s) (CVD), including prior myocardial infarction, myocardial and/or revascularisation procedures, stroke]; *(iii)* medications before admission (antihypertensive drugs, antiplatelet therapy, anticoagulants, statins and anti-diabetes agent); *(iv)* symptoms (fever, dyspnea, cough, fatigue, diarrhea or atypical symptoms) and *(v)* laboratory parameters at admission [fasting plasma glucose (FPG), full blood count, renal function and electrolytes, hepatic profile, lactate dehydrogenase (LDH), creatine kinase (CK), inflammatory markers, including c-reactive protein (CRP), Procalcitonin (PCT), fibrinogen and D-dimer, and coagulation]; *vi* blood gas analysis parameters, including the PaO_2_/FiO_2_ ratio (P/F), as indicator of hypoxemia severity [20]; *vii* electrocardiographic (EKG) abnormalities. eGFR (estimated glomerular filtration rate) was calculated using the Chronic Kidney Disease Epidemiology Collaboration (CKD-EPI) formula [21]. DM was defined for glucose levels in accordance with the American Diabetes Association Criteria and FPG was examined both as continuous variable and as lower/higher than 126 mg/dl as a cut-off value for the DM range [22]. Data derived from chest HRCT were obtained according to a published protocol [23]. Data on in-hospital management including oxygen therapy -nasal cannulas (NC), Venturi Mask (VM), reservoir (RV), non-invasive ventilation (NIV)-, antivirals and antibiotics, prophylaxis/antithrombotic therapy, immunosuppressants use, were also recorded.

### Study outcomes

Primary endpoint was the cumulative incidence rate of the composite endpoint of in-hospital death/transfer to ICU in patients with known-DM compared to those without or unknown-DM. Secondary endpoints included the cumulative incidence rates in subjects with known-DM compared to those without or unknown-DM of *i* death *ii* transfer to ICU *iii*. length of stay, as well as *iv*.the prognostic factors for composite endpoint in hospitalized patients.

### Statistical analysis

Categorical variables were described as proportions (%), and continuous variables were reported as median and interquartile range (IQR). Comparisons between groups were run by Student’s t test (normally distributed data) or Mann-Whitney test (non-normally distributed data). Proportions for categorical variables were compared using the χ2 test. Univariate and multivariate logistic regression models were used to identify independent risk factors associated with primary endpoint. To assess the overall predictive power of the different regression models ROC curves and area under the curve (AUC) were calculated. The cumulative proportion of patients free from the composite primary endpoint in people with and without DM was plotted by applying Kaplan-Meier method. Statistical significance was declared at two-sided *p* < 0.05. Analyses were performed with SPSS 27 (IBM Statistics).

## Results

### Study population

**Fig. 1:**
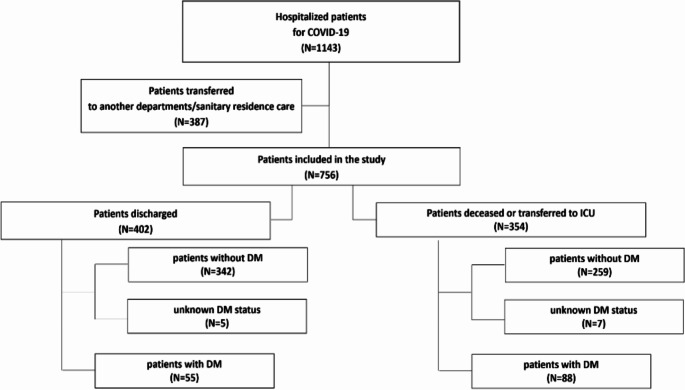
Flow diagram of the study. DM, diabetes mellitus; ICU, intensive care unit

Between 23^rd^ February 2020 and 31^st^ March 2020, 1143 adult patients were admitted to the Covid-1 macro-unit of the University Hospital of Parma, Italy. The study flow diagram is shown in Fig. 1. After excluding patients transferred to other departments or sanitary residence care (*n* = 387; 33.9%), 756 (66.1%) inpatients were included in the study. Of these, 402 (53.2%) were discharged from hospital and 354 (46.8%) transferred to ICU (*n* = 56, 7.4%) and/or deceased (*n* = 298, 39.4%). Known-DM was present in 143 patients (18.9%), of whom 55 were in the discharged group (13.7%) and 88 (24.8%) were in the group meeting the primary endpoint (transferred to ICU or deceased). In 12 patients admitted with hyperglycemia (1.6%; respectively, 5 and 7 patients in the discharged and deceased/transferred to ICU groups), the diagnosis of DM could not be confirmed due to data missing, and the subjects were classified as having an “unknown-DM status”.

### Study population characteristics

The main demographic and clinical characteristics of the study subjects according to DM status are described in Table 1. Subjects admitted with COVID-19 had a mean age of 70 years and were predominantly males (61.1%); individuals with DM were older (*p* < 0.001) and showed a higher prevalence of male gender (*p* = 0.04) than individuals with no/unknown-DM.

BMI did not differ between groups (*p* = 0.07), although the prevalence of obesity (BMI ≥ 30) was significantly higher in subjects with DM compared to those without (40.6% vs. 33.0%, *p* = 0.03).

Main comorbidities in admitted subjects were hypertension (61.2%), followed by cancer (15.7%), COPD (10.5%), atrial fibrillation (10.2%), CHF (6.7%) and CKD (6.5%). Notably, higher rates of hypertension, CHF, CKD (all *p* < 0.01) were present in subjects with DM compared to those without/unknown-DM.

**Table 1:** Demographic and clinical characteristics of study subjects

Characteristic			Missing data	All *n* = 756	No DM/ Unknown-DM *n* = 613	DM *n* = 143	*P* value
*Age (year)*			0	70 (59–80)	69 (58–80)	76 (66–82)	< 0.001
*Male sex*			0	462 (61.1)	364 (59.4)	98 (68.5)	0.04
*Body mass index (kg/m* ^*2*^ *)*	*< 25*		63	106 (14.02)	87 (14.2)	19 (13.3)	0.07
	*25–29.9*			327 (43.2)	278 (45.3)	49 (34.3)	
	*≥ 30*			260 (34.4)	202 (33.0)	58 (40.6)	
*Coexisting conditions*	*Hypertension*		13	465 (61.2)	345 (56.3)	120 (83.9)	< 0.001
	*AF*		20	77 (10.2)	55 (9.0)	22 (15.4)	0.03
	*CHF*		21	50 (6.7)	26 (4.2)	24 (6.8)	< 0.001
	*CDK stage 3*		0	231 (30.6)	158 (258)	73 (14.0)	0.005
	*Cancer*		16	119 (15.7)	92 (15.0)	27 (18.9)	0.34
	*COPD*		17	79 (10.5)	59 (9.6)	20 (14.0)	0.34
*History of CVD*			26	186 (24.6)	123 (20.1)	63 (44.1)	< 0.001
*Length of stay (days)*			0	6 (3–11)	6 (3–11)	7 (4–11)	0.44
*Death*			0	333 (44.0)	249 (40.6)	84 (58.7)	< 0.001

Age and length of stay are expressed as median (IQR), other data are given as number (%). AF, Atrial fibrillation; CHF chronic heart failure; CDK stage 3, chronic kidney disease (defined as eGFR < 60 mL/min/1.73 m^2^); COPD, chronic obstructive pulmonary disease; CVD, cardiovascular disease.

Nearly 25% of the subjects experienced a previous CVD, with double prevalence in DM (44%) vs. no/unknown-DM (20%) individuals (*p* < 0.001).

In-hospital mortality rate was significantly higher in patients with DM, in comparison with those without (*p* < 0.001). Median length of stay was 6 days.

In accordance with the comorbidity distribution, the main chronic drug treatments were antihypertensives (61.5%), mainly ACE inhibitors and angiotensin receptor blockers (ARBs), antiplatelet agents (24.1%), statins (24.1%) and anti-coagulants (12.0%). Individuals with DM showed a higher frequency of administration of all drug classes (*p* < 0.001), except for anti-coagulants (*p* = 0.13). As for glucose lowering agents, there was a higher prevalence of metformin use (56.6%) followed by insulin (27.3%), sulfonylureas (14.7%) and others. Medication use at admission is shown in Supplementary table SI1.

Most common SARS-CoV-2 symptoms at admission are shown in Supplementary table SI2. Fever was present in 81.9% of the subjects followed by cough and dyspnea in nearly half of the patients. These symptoms were more frequently recorded in individuals with no/unknown-DM compared to those with DM (84.2% vs. 72.0%, *p* < 0.001 for the fever and 51.7% vs. 41.3%, *p* = 0.03 for cough). EKC abnormalities were significantly higher in subjects with DM compared to no/unknown-DM (51.7% vs. 40.3%, *p* = 0.001).

Oxygen saturation (measured through finger pulse oximeter) at admission was lower in patients with DM (90% vs. 93% with no/unknown-DM, *p* < 0.001, data not shown) along with P/F ratio (*p* < 0.001) The CT-visual score (percentage of lung parenchymal involvement) at admission was 30% in subjects without compared to 35% in those with DM (*p* = 0.38).

Table 2 shows lab parameters at admission. Beside blood glucose levels (*p* < 0.001), also urea, creatinine and potassium levels (all *p* < 0.001) were higher in individuals with DM vs. no/unknown-DM. Of note, eGFR was significantly lower in DM compared to no/unknown-DM individuals (*p* < 0.001). Similarly, subjects with DM had higher levels of D-dimer (*p* = 0.03), red cell distribution width (RWD, *p* = 0.03), and PCT (*p* = 0.001). No significant differences were found in white blood cell (WBC) count, liver enzymes and pancreatic lipase, CK, LDH and CRP.

**Table 2:** Laboratory parameters at admission

Characteristic	Missing data	All *n* = 756	No DM/ Unknown-DM *n* = 613	DM *n* = 143	*p* value
*WBC– x10* ^*3*^ */µl*	15	6.91 (5.11–9.47)	6.90 (4.97–9.33)	7.01 (5.65–9.85)	0.096
*Lymphocytes– x10* ^*3*^ */ µl*	17	0.90 (0.63–1.24)	0.9 (0.63–1.25)	0.95 (0.65–1.21)	0.78
*Neutrophils– x10* ^*3*^ */ µl*	18	5.46 (3.71–7.91)	5.42 (3.63–7.86)	5.79 (4.19–8.27)	0.07
*Monocytes– x10* ^*3*^ */ µl*	18	0.42 (0.27–0.60)	0.42 (0.27–0.60)	0.42 (0.29–0.63)	0.75
*NLR*	19	5.89 (3.41–10.65)	5.80 (3.30–10.65)	6.10 (3.91–10.72)	0.19
*PLT– x10* ^*3*^ */ µl*	13	207 (164–269)	207 (162–265)	207 (173–290)	0.17
*RWD– fl.*	288	44.4 (41.7–47.4)	44.2 (41.6–47.1)	45.1 (42.5–49.3)	0.03
*Hb– g/ µl*	13	13.6 (12.4–14.7)	13.7 (12.5–14.7)	13.4 (11.8–14.7)	0.05
*Urea– mg/dl*	20	44 (30.2–67)	41 (30–61)	59 (40–96)	< 0.001
*Creatinine– mg/dl*	16	0.9 (0.7–1.2)	0.9 (0.7–1.1)	1.1 (0.9–1.7)	< 0.001
eGFR– ml/min*1.73 m ^*2*^	16	79.8 (53.7–93.8)	82.7 (58.8–95.5)	59.5 (34.1–82.2)	< 0.001
*Sodium– mEq/l*	20	137 (135–140)	137 (135–140)	137 (135–139)	0.13
*Potassium– mEq/l*	20	4.0 (3.7–4.3)	4.0 (3.7–4.3)	4.2 (3.8–4.6)	< 0.001
*FPG ≥ 126 mg/dl*	199	210(27.8)	128(20.9)	82(57.3)	< 0.001
*FPG– mg/dl*	199	115 (98–143)	109 (96–130)	180 (129–249)	< 0.001
*AST– U/*	58	47 (33–73)	48 (34–73)	46 (30–70)	0.34
*ALT– U/L*	41	32 (21–50)	32 (22–51)	30 (21–47)	0.35
*Total Bilirubin– mg/dl*	43	0.7 (0.5–0.9)	0.7 (0.5–0.9)	0.7 (0.5–0.8)	0.32
*LDH– U/L*	66	370 (282–502)	367 (282–499)	384 (293–525)	0.41
*CK– U/L*	69	139 (77–324)	138 (77–307)	142 (82–344)	0.50
*D-dimer– ng/ml*	231	931 (613–1627)	877 (604–1542)	1075 (695–2022)	0.03
*CRP– mg/l*	55	105.6 (51.9-167.7)	104 (50–166)	119 (64–178)	0.08
*PCT– ng/ml*	117	0.19 (0.08–0.56)	0.17 (0.08–0.49)	0.32 (0.10–0.88)	0.001
*Fibrinogen– mg/dl*	142	629 (502–765)	629 (513–779)	612 (502–754)	0.80
*PT/INR*	160	1.21 (1.14–1.33)	1.21 (1.14–1.34)	1.21 (1.14–1.33)	0.47
*INR*	197	1.21 (1.14–1.33)	1.21 (1.14–1.33)	1.22 (1.14–1.34)	0.28

Data are given as mean ± SD or median (interquartile range). WBC, white blood cells; NLR, neutrophil to lymphocyte ratio; PLT, platelet count; RWD, Red Cell Distribution Width; Hb, Hemoglobin; eGFR, estimated glomerular filtration rate; FPG, fasting plasma glucose; AST, aspartate aminotransferase; ALT, alanine transaminase; LDH, Lactate dehydrogenase; CK, creatine kinase; CRP, C-reactive protein; PCT, Procalcitonin; PT, Prothrombin; INR, International Normalized Ratio

According to the hospital COVID-19 treatment protocol in force during the first pandemic wave, the most common drugs used during hospitalization were: antibiotics (mostly: 78% ceftriaxone, 77% azithromycin and 73% ceftriaxone + azithromycin), immunosuppressants (53% hydroxychloroquine), antivirals (mostly association of darunavir and ritonavir, 29%). Anticoagulants and steroid therapies were introduced only in the late phase of the first wave. No significant differences in treatments were found by DM status. Oxygen therapy at admission was more frequent in subjects with DM (*p* < 0.002), with no difference in O2 delivery device (Supplementary Table SI3).

### Composite primary endpoint

During the observation period 354 events occurred, including 300 (85%) deaths and 54 (15%) ICU transfers, of which 33 (61%) subsequently deceased.

**Fig. 2:**
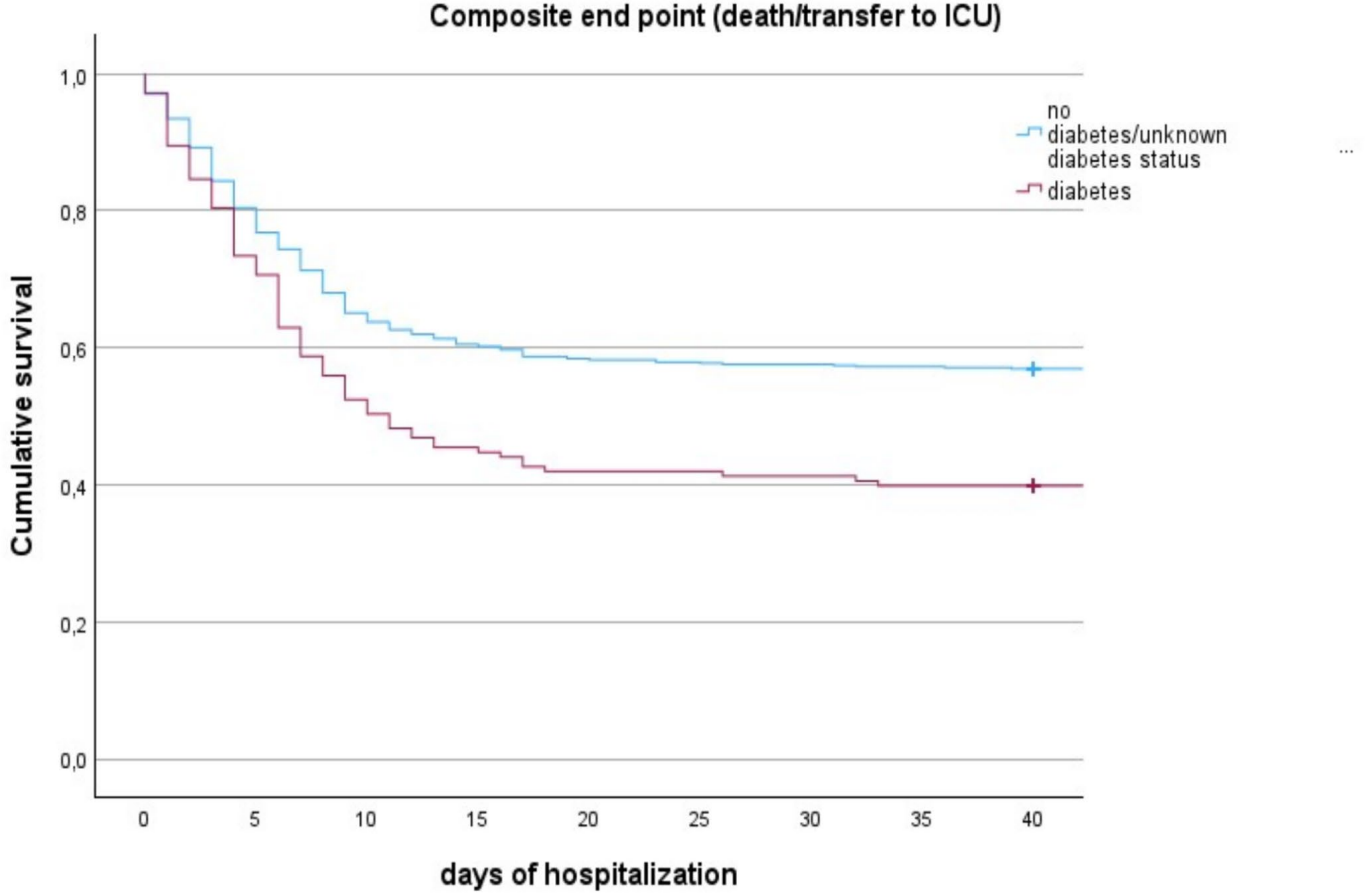
Kaplan–Meier curve for cumulative survival considering mortality/ICU admission during hospitalization

Among the 354 events, 88 (61.5%) occurred in patients with DM and 266 (43.4%) in no/unknown-DM (*p* < 0.001) (Fig. 2). The estimated mean time to endpoint was 25 ± 3 days in subjects with DM and 36 ± 6 days in those without DM (log-rank test *p* < 0.001).

### Secondary endpoints

During the observation period 81 (56.6%) death in DM vs. 219 (35.7%) in unknown/no diabetes individuals occurred (*p* < 0.001) (Supplementary figure SI1A). Conversely, no differences were observed in the incidence of transfer to ICU in DM (7, 4.9%) vs. unknown/no diabetes (47, 7.7%) (*p* = 0.42) (Supplementary figure SI1B). The mean time to transfer was 50 ± 5 vs. 66 ± 1 (*p* = 0.31). No differences in length of stay were observed between study groups with a median time of 7 (IQR:4–11) in DM and 6 (IQR: 3–11) in unknown/no diabetes (*p* = 0.42).

Significant predictors of composite endpoint identified at univariate analysis included age, previous CV event, DM status, BMI ≥ 30 kg/m^2^, FPG at admission, arterial hypertension, CHF, COPD, CT-visual score, treatment with insulin, ASA, beta-blockers, statin, dyspnea at admission, EKG abnormalities, P/F ratio, WBC, NRL, RDW, LDH, CRP, D-dimer, PCT, and eGFR (data not shown). After including the most significant variables associated with primary outcome and taking into account the collinearity among predictors in the multivariate regression model, a previous diagnosis of DM was not independently associated with the primary endpoint (*p* = 0.25). However, FPG in the diabetic range (≥ 126 mg/dL), along with older age, previous CV events, CKD, CRP, and P/F ratio were confirmed to be independent risk indicators of death and/or transfer to ICU as reported in Table 3.

**Table 3:** Multivariate logistic regression for composite primary endpoint

Variable	No.	OR	95% CI	*P*-val
*Age– years*	431	1.076	1.046–1.106	< 0.001
*Previous CVD*	431	3.799	1.768–8.163	< 0.001
*P/F*	431	0.988	0.984–0.991	< 0.001
eGFR < 60 ml/min*1.73 m ^*2*^	431	2.828	1.359–5.885	0.005
*FPG ≥ 126 mg/dl*	431	1.958	1.050–3.651	0.034
*CRP*	431	1.005	1.001–1.010	0.019

Multivariate logistic regression for composite primary endpoint adjusted for age, previous cardiovascular disease (CVD), estimated glomerular filtration rate (eGFR) < 60 mL/min*1.73m^2^, fasting plasma glucose (FPG) ≥ 126 mg/dl, C-reactive protein (CRP) and PaO_2_/FiO_2_ (P/F) ratio. OR, odds ratio; CI, confidence interval

Since FPG in the diabetic range (≥ 126 mg/dL) was identified as an independent risk indicator in the multivariate model, the analysis of the primary endpoint was also conducted stratifying by FPG levels. The cumulative survival rate differed significantly between patients with FPG below and above 126 mg/dL (log-rank test *p* < 0.001) (Supplementary Figure SI2), with an estimated mean time to event of 36 ± 6 days and 21 ± 2 days, respectively.

**Fig. 3:**
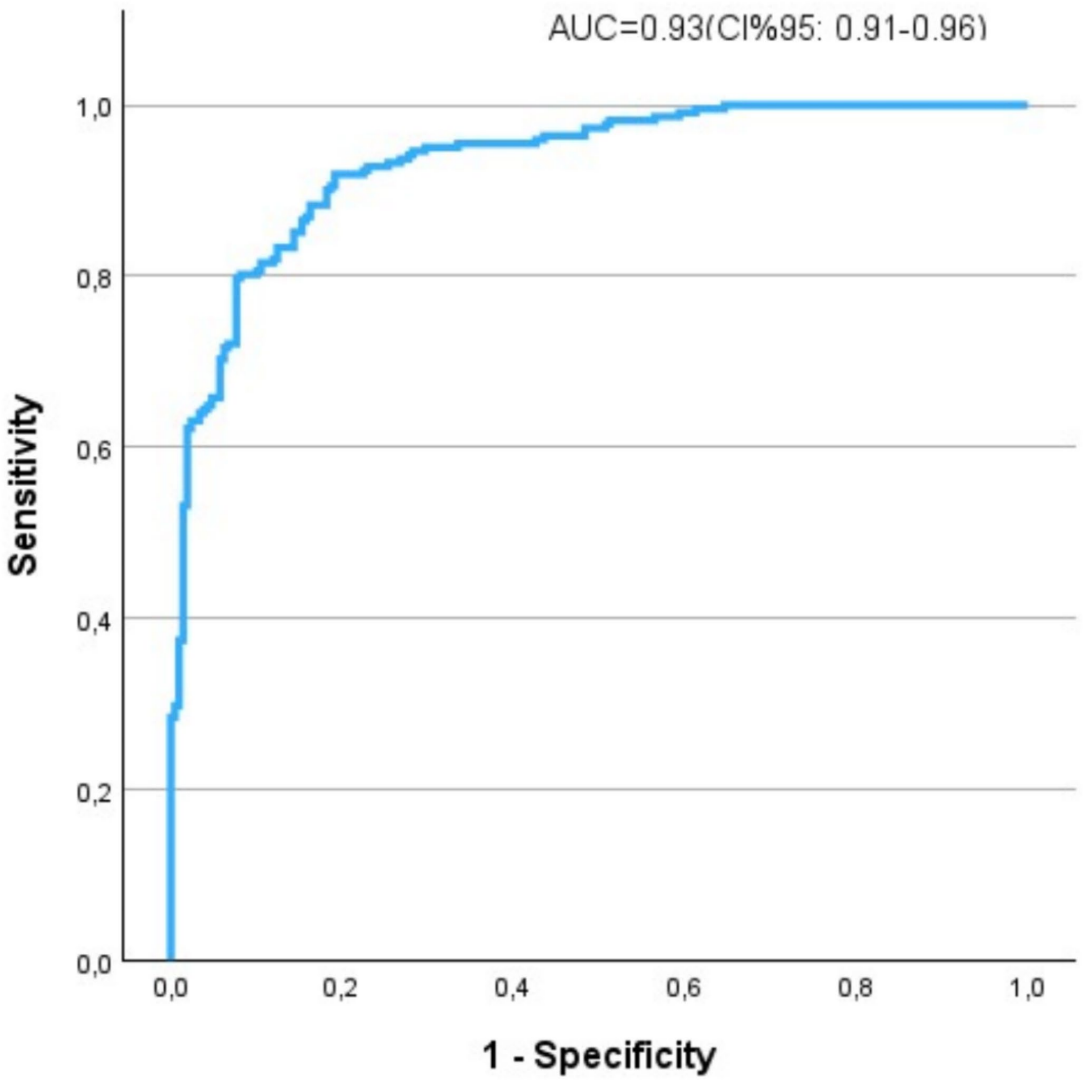
ROC curve for final logistic model. The model included age, previous cardiovascular disease (CVD), estimated glomerular filtration rate (eGFR) < 60 mL/min*1.73m^2^, fasting plasma glucose (FPG) ≥ 126 mg/dl, C-reactive protein (CRP) and PaO_2_/FiO_2_ (P/F) ratio as predictors

The ROC curve obtained from probabilities of the final model confirmed a very good accuracy in predicting the composite endpoint, as shown in Fig. 3.

## Discussion

In this study, the high adverse outcome rates in individuals hospitalized for COVID-19 should be contextualized in the rapid progression of COVID-19 and the high burden on healthcare systems during the first wave due to the overwhelming number of cases and lack of treatment options with proof of efficacy at the time. In a large meta-analysis, the pooled prevalence of mortality among hospitalized patients with COVID-19 was 17.62% with COPD, CVD, DM, hypertension, obese, cancer, acute kidney injury and increase D-dimer being main risk factors for increased mortality [24].

We selected only consecutive patients hospitalized for severe COVID-19 during the first pandemic phase with exclusive clinical management in the Covid-1 macro-unit of the University Hospital of Parma. The reason to our restrict attention to this time/place was an attempt to limit the confounding role(s) played by the large diversity in clinical management often present in previously published cohorts, due to different clinical settings and protocols and different availability and use of proven efficacious therapies, e.g. dexamethasone. As a result, it should be easier to isolate the relevant biologic factors, and especially DM, heralding, and perhaps underlying, poor outcome in patients with severe COVID-19.

The prevalence of DM in our cohort (18.9%) is notably lower than the figures reported in the literature and Italian epidemiological data, where prevalence rates are approximately 30% [7, 15]. In our cohort, individuals with DM experienced a significantly poorer prognosis compared to those without known-DM, aligning with existing literature [25, 26]​. In accordance with our study, the relative risk of mortality was 1.75 greater in individuals with DM compared to the general population who died with SARS-CoV-2 infection [5, 27]. Advanced age was pointed as a major confounder of the role played by DM per se in the prognosis of patients with severe COVID-19 [28].

In our cohort, individuals with DM were older with a higher male prevalence with a greater rate of obesity and coexisting conditions including hypertension, CHF, CKD and notably previous CVD history all well-known harbingers of poor COVID-19 prognosis [29, 30].

Of note, in critically ill patients with DM, poor glycemic control leads to an increased mortality rate, infection rate, mechanical ventilation, and prolonged hospitalization [31]. In our study, people with known-DM also displayed more frequently higher levels of FPG, D-dimer and PCT with lower PaO2/FiO2 without differences in CT visual scores of lung parenchymal involvement, suggesting greater functional damage than in non-DM. This pattern raises the question of whether DM itself, independently of hyperglycemia, or both, accounts for the higher risk of poor COVID-19 outcomes in these patients.

In our study, FPG in the diabetic range at admission, but not known-DM, was an independent risk indicator of poor COVID-19 outcome. This condition embraces DM, undiagnosed DM and stress hyperglycemia, which may be due to several factors, including, but not limited to, increased release of stress hormones and other biochemical changes, which cause/exacerbate insulin-resistance [32–34]. In addition, COVID-19 may have a diabetogenic effect independent of the stress response during a severe disease due to the virus-induced β-cell cytotoxicity leading to an increased rate of new-onset DM [35, 36].

The new-onset hyperglycemia caused by COVID-19 resulted in long-term hyperglycemia, worse clinical outcomes, prolonged hospital stays, and a greater need for oxygen support or positive pressure ventilation [36]. Newly detected DM was a powerful predictor of poor outcomes [15, 37]. Our findings are consistent with prior literature indicating that, in patients with acute medical conditions, FPG ≥ 7.0 mmol/l (126 mg/dl) at admission is independently associated with adverse clinical outcomes. Diabetic fasting hyperglycemia was an independent predictor for 28-day mortality in patients with COVID-19 without previous diagnosis of DM [37]. However, HbA1c was not systematically measured in our cohort, and unknown-DM remains a potential confounder of our results.

As to stress hyperglycemia is common in people with severe infections, and reflects a number of underlying biological processes, which may have a detrimental impact of their own, on the patient outcome and it is the most likely explanation for the different prevalence of known-DM and FPG in the diabetic range in our cohort. Systematic reviews have reported that high FPG on hospital admission for COVID-19 was associated with increased risk of ICU transfer and death [38].

Hyperglycemia is associated with increased cytokine storm - IL-6 and CRP levels - and immune dysfunction - decreased lymphocytes, and T cells - in COVID-19 [39].

In a large cohort of COVID-19 patients without pre-existing metabolic-related diseases, insulin-resistance was retrospectively pointed as the direct cause of hyperglycemia. Mechanistically, SARS-CoV-2 infection modulates the expression of some secreted metabolic factors resulting in the perturbation of glucose metabolism [40].

However, the bidirectional relationship of acute infection and hyperglycemia makes it difficult to draw conclusions as to whether poor outcomes reflect the effect of acute/chronic hyperglycemia on COVID-19 severity or acute hyperglycemia simply is a byproduct of the severity of infection.

It is noteworthy that, among the independent indicators of poor COVID-19 outcome selected by multivariable analysis, hyperglycemia can be considered an actionable target of therapy. Hyperglycemia could and should be treated to reach and maintain the levels associated with the best prognosis [31]. To this regard it should be noted that, at variance with various large studies conducted in people hospitalized in the pre-COVID-19 era, people with best prognosis in this study had FPG < 7 mmol/l (126 mg/dl) [41].

Since known-DM was not included in the multivariate analysis, the impact of DM on poor COVID-19 prognosis may be driven mostly by concomitant factors and complications, as already reported [42]. On the other hand, the fact that, beyond DM, also obesity and hypertension were not predictors of poor outcome may hint that the statistical power of our cohort was insufficient to gauge their independent role.

In addition to age and hyperglycemia, in accordance with the literature, cardio-renal burden and the degree of functional respiratory impairment in terms of P/F ratio were independent factors of poor outcome explaining approximatively 95% of the primary endpoint [30, 43]. These patients should, therefore, be identified and receive early intervention and treatment to prevent mortality in course of COVID-19 infection and possibly, further evidence is needed, of other future respiratory viral infection.

## Conclusions

Known-DM was associated with poor COVID-19 outcomes, but not when adjusted for other baseline clinical variables (e.g. age, hypertension and obesity) and comorbidities (previous CV event, history of CKD), suggesting that the impact of DM was mostly driven by concomitant factors and complications. Diabetic fasting hyperglycemia was associated with COVID-19 severity in fully adjusted analyses, representing a powerful and independent predictor of poor outcomes, with a stronger association than pre-existing DM. However, whether these patients had undiagnosed DM, newly-onset DM or stress hyperglycemia remains unclear. The insights gained from COVID-19 research and data may have several significant implications for the management and treatment of DM hyperglycemia not only limited to the SARS-CoV2 infection but also to other respiratory infectious diseases.

## Electronic supplementary material

Below is the link to the electronic supplementary material.

Supplementary Material 1Supplementary Material
