# Supplementary material for: Hyperglycemia in the diabetic range, but not previous diagnosis of diabetes mellitus, is an independent indicator of poor outcome in patients hospitalized for severe COVID-19
Source: Acta Diabetol. 2025 May 2;62(10):1751–60. doi: 10.1007/s00592-025-02507-1 (PMC12578703; doi:10.1007/s00592-025-02507-1)
Supplement: Supplementary file 1 — Supplementary Material 1Supplementary Material [file 592_2025_2507_MOESM1_ESM.docx]

***Supplementary Information***

***Methods***

*Data collection*

Data were retrieved from inpatient medical records including *i.* demographic data (age and gender), *ii.* past medical history [hypertension, atrial fibrillation (AF), renal (chronic kidney disease, CKD) or heart (congestive heart failure, CHF) failure, cancer, chronic obstructive pulmonary disease (COPD), previous cardiovascular event(s) (CVD), including prior myocardial infarction, myocardial and/or revascularisation procedures, stroke]; *iii.* medications before admission (antihypertensive drugs, antiplatelet therapy, anticoagulants, statins and anti-diabetes agent); *iv.* symptoms (fever, dyspnea, cough, fatigue, diarrhea or atypical symptoms) and *v.* laboratory parameters at admission [fasting plasma glucose (FPG), full blood count, renal function and electrolytes, hepatic profile, lactate dehydrogenase (LDH), creatine kinase (CK), inflammatory markers, including c-reactive protein (CRP), Procalcitonin (PCT), fibrinogen and D-dimer, and coagulation]; *vi* blood gas analysis parameters, including the PaO_2_/FiO_2_ ratio (P/F), also known as the Horowitz index, as indicator of hypoxemia severity [20]. eGFR (estimated glomerular filtration rate) was calculated using the Chronic Kidney Disease Epidemiology Collaboration (CKD-EPI) formula [21]. DM was defined for glucose levels in accordance with the American Diabetes Association Criteria and FPG was examined both as continuous variable and as lower/higher than 126 mg/dl as a cut-off value for the DM range [22]. Electrocardiographic (EKG) abnormalities [left ventricular hypertrophy (LVH), atrioventricular (AV) conduction abnormalities (complete or second-degree AV block), pacemaker, atrial fibrillation (AF), isolated Q/QS-wave abnormalities, incomplete (left or right) bundle branch block (RBBB or LBBB), left-axis deviation (LAD), isolated major ST/T-wave abnormalities] were recorded.

Data derived from chest HRCT were obtained according to a published protocol [23]. Briefly, the overall extent of interstitial pneumonia was assessed according to a "visual score" developed in the Radiology Division of this University Hospital as previously described [24], [25]; clinical outcomes, including length of stay (days), transfer to other clinical units, destination of discharge (home/sanitary residence care); and death/transfer to ICU, were also collected. Height and weight to calculate body mass index (BMI) were not available in most medical records: they were estimated by using neck circumference/diameters and thoracic spine length, as measured by HRCT, according to an equation developed and validated in two independent cohorts (discovery and replication) of patients (data not shown) [26].

Data on in-hospital management including oxygen therapy -nasal cannulas (NC), Venturi Mask (VM), reservoir (RV), non-invasive ventilation (NIV)-, antivirals and antibiotics, prophylaxis/antithrombotic therapy, immunosuppressants use, were also recorded.

**References**

[20] The ARDS Definition Task Force*, «Acute Respiratory Distress Syndrome: The Berlin Definition», JAMA, vol. 307, fasc. 23, pp. 2526–2533, giu. 2012, doi: 10.1001/jama.2012.5669.

[21] G. Pugliese et al., «The Chronic Kidney Disease Epidemiology Collaboration (CKD-EPI) equation provides a better definition of cardiovascular burden associated with CKD than the Modification of Diet in Renal Disease (MDRD) Study formula in subjects with type 2 diabetes», Atherosclerosis, vol. 218, fasc. 1, pp. 194–199, set. 2011, doi: 10.1016/j.atherosclerosis.2011.04.035.

[22] American Diabetes Association, «2. Classification and Diagnosis of Diabetes: Standards of Medical Care in Diabetes-2021», Diabetes Care, vol. 44, fasc. Suppl 1, pp. S15–S33, gen. 2021, doi: 10.2337/dc21-S002.

[23] D. Colombi et al., «Well-aerated Lung on Admitting Chest CT to Predict Adverse Outcome in COVID-19 Pneumonia», Radiology, vol. 296, fasc. 2, pp. E86–E96, ago. 2020, doi: 10.1148/radiol.2020201433.

[24] D. Colombi et al., «Qualitative and quantitative chest CT parameters as predictors of specific mortality in COVID-19 patients», Emerg. Radiol., vol. 27, fasc. 6, pp. 701–710, dic. 2020, doi: 10.1007/s10140-020-01867-1.

[25] D. Colombi et al., «Quantitative CT at Follow-Up of COVID-19 Pneumonia: Relationship with Pulmonary Function Tests», Diagn. Basel Switz., vol. 13, fasc. 21, p. 3328, ott. 2023, doi: 10.3390/diagnostics13213328.

[26] N. Namazi, B. Larijani, P. J. Surkan, e L. Azadbakht, «The association of neck circumference with risk of metabolic syndrome and its components in adults: A systematic review and meta-analysis», Nutr. Metab. Cardiovasc. Dis. NMCD, vol. 28, fasc. 7, pp. 657–674, lug. 2018, doi: 10.1016/j.numecd.2018.03.006.

**Table SI1.** Concomitant therapies at admission by DM status.

| Agents | | | | | Missing data | All  n=756 | No DM/  Unknown DM  n=613 | DM  n=143 | P value |
| --- | --- | --- | --- | --- | --- | --- | --- | --- | --- |
| ***Glucose-lowering drugs*** | | | |  | 19 | 117 (15.5) | 0 (0.0) | 117 (100.00) | <0.001 |
|  | *Metformin* | | | |  | 81 (10.7) | 0 (0.0) | 81 (56.6) | <0.001 |
|  | *Sulfonylurea* | | | |  | 21 (2.8) | 0 (0.0) | 21 (14.7) | <0.001 |
|  | *Insulin* | | | |  | 39 (5.2) | 0 (0.0) | 39 (27.3) | <0.001 |
|  | *DPP4 inhibitors* | | | |  | 18 (2.4) | 0 (0.0) | 18 (12.6) | <0.001 |
|  | *Pioglitazone* | | | |  | 3 (0.4) | 0 (0.0) | 3 (2.1) | <0.001 |
|  | *SGLT2 inhibitors* | | | |  | 9 (1.2) | 0 (0.0) | 9 (6.3) | <0.001 |
|  | *GLP-1RA* | | | |  | 4 (0.9) | 0 (0.0) | 4 (2.8) | <0.001 |
| ***Antihypertensive drugs*** | | |  | | 13 | 465 (61.5) | 345 (56.3) | 120 (83.9) | <0.001 |
|  | *ACE inhibitors, ARBs* | | | |  | 293 (38.8) | 213 (35.4) | 80 (55.9) | <0.001 |
|  | *Diuretics* | | | |  | 192 (25.4) | 131 (21.8) | 61 (42.7) | <0.001 |
|  | *Beta-blockers* | | | |  | 227 (30.0) | 156 (26.0) | 71 (49.7) | <0.001 |
|  | *Calcium channel blockers* | | | |  | 152 (20.4) | 101 (16.8) | 51 (35.7) | <0.001 |
|  | *Alpha-blockers* | | | |  | 56 (7.5) | 37 (6.2) | 19 (13.3) | 0.01 |
| ***ASA*** |  | | | | 38 | 182 (24.1) | 116 (18.9) | 66 (46.2) | <0.001 |
| ***Anticoagulants*** | |  | | | 30 | 91 (12.0) | 68 (11.1) | 23 (16.1) | 0.13 |
| ***Statins*** |  | | | | 30 | 182 (24.1) | 116 (18.9) | 66 (46.2) | <0.001 |

Data are given as number (%). DPP4, dipeptidyl peptidase-4; SGLT2, sodium-glucose cotransporter-2; GLP-1RA, glucagon-like peptide-1 receptor agonist; ACE, angiotensin-converting enzyme; ARBs, angiotensin receptor blockers; ASA, acetylsalicylic acid

**Supplementary table SI 2**. **Clinical and diagnostic setting at admission.**

| Characteristic | | Missing data | All  No. 756 | No DM/  Unknown DM  No. 613 | DM  No. 143 | P value |
| --- | --- | --- | --- | --- | --- | --- |
| *Symptoms at admission* | | 41 |  |  |  |  |
|  | *Fever* |  | 619 (81.9) | 516 (84.2) | 103 (72.0) | 0.001 |
|  | *Cough* |  | 376 (49.7) | 317 (51.7) | 59 (41.3) | 0.03 |
|  | *Dyspnea* |  | 374 (49.5) | 296 (48.3) | 78 (54.5) | 0.075 |
|  | *Fatigue* |  | 75 (10.0) | 56 (9.3) | 19 (13.3) | 0.05 |
|  | *Diarrhea* |  | 42 (5.5) | 33 (5.4) | 9 (6.3) | 0.44 |
|  | *Atypical symptoms* |  | 107 (14.4) | 86 (14.3) | 21 (14.7) | 0.32 |
| *Chest CT score – %* | | 41 | 30 (15-50) | 30 (15-50) | 35 (15-50) | 0.38 |
| *PaO_2_/FiO_2_* | | 135 | 202 (93-309) | 215 (94-314) | 161 (83-275) | <0.001 |
| *Abnormal EKG* | | 86 | 321 (42.5) | 247 (40.3) | 74 (51.7) | 0.001 |

Data are given as number (%) for categorical variables and as interquartile ranges for continuous variables.

CT, computed tomography; P/F, PaO_2_/FiO_2_ ratio; EKG, electrocardiogram.

**Supplementary Table SI3**. Therapies during hospitalization

| **Therapy** | **missing** | **All**  **n=756** | **No DM/**  **Unknown DM**  **n=613** | **DM**  **n=143** | **p-value** |
| --- | --- | --- | --- | --- | --- |
| Steroids | 35 | 139(18.4) | 110(17.9) | 29(20.3) | 0.56 |
| Anticoagulants | 44 | 49(6.5) | 44(7.2) | 5(3.5) | 0.12 |
| Ceftriaxone+azithromycin | 30 | 545(72.1) | 444(72.4) | 101(70.6) | 0.81 |
| Ceftriaxone | 26 | 585(77.4) | 475(77.5) | 110(76.9) | 0.81 |
| Azithromycin | 26 | 577(76.3) | 470(76.7) | 107(74.8) | 0.91 |
| Piperacillin+tazobactam | 30 | 141(18.6) | 111(18.1) | 30(21.0) | 0.39 |
| Linezolid | 29 | 117(15.7) | 96(15.7) | 21(14.7) | 0.82 |
| Levofloxacin | 41 | 43(5.7) | 34(5.5) | 9(6.3) | 0.67 |
| Amoxicillin/clavulanic acid | 41 | 40(5.3) | 34(5.5) | 6(4.2) | 0.54 |
| Azithromycin +chloroquine | 30 | 56(7.4) | 48(7.8) | 8(5.6) | 0.55 |
| Chloroquine | 26 | 398(52.6) | 326(53.2) | 72(50.3) | 0.75 |
| Colchicin | 28 | 70(9.4) | 59(9.6) | 11(7.7) | 0.74 |
| Lopinavir+ritonavir | 26 | 172(22.8) | 134(21.9) | 38(26.6) | 0.43 |
| Darunavir+ritonavir | 29 | 220(29.1) | 183(29.9) | 37(25.9) | 0.61 |
| Tocilizumab | 33 | 32(4.3) | 28(4.6) | 4(2.8) | 0.61 |
| Supplemental oxygen therapy at admission  -nasal cannulas (NC)  -venturi mask (VM)  -reservoir (RV | 17 | 498(66.1)  204(27.0)  58(7.7)  236(31.2) | 389(63.5)  159(25.9)  42(6.9)  188(30.7) | 109(76.2)  45(31.5)  16(11.2)  49(34.3) | 0.002  0.16  0.08  0.39 |

Data are expressed as number (%). OR, odds ratio; CI, confidence interval. History of CVD. DM, Diabetes mellitus; AF, atrial fibrillation; CHF, chronic heart failure; COPD, chronic obstructive pulmonary disease; ASA, acetylsalicylic acid; EKG, electrocardiogram; CT, computed tomography; P/F, PaO_2_/FiO_2_ ratio; FPG, fasting plasma glucose; WBC, white blood cells; NLR; neutrophil to lymphocyte ratio; RDW, red cell distribution width; eGFR, estimated glomerular filtration rate; LDH, lactate dehydrogenase; CRP, C-reactive protein; PCT, procalcitonin.

**Figure SI1.** Cumulative survival rates for death(A) and ICU admission(B) during hospitalization according to diabetes status.

**A**

**
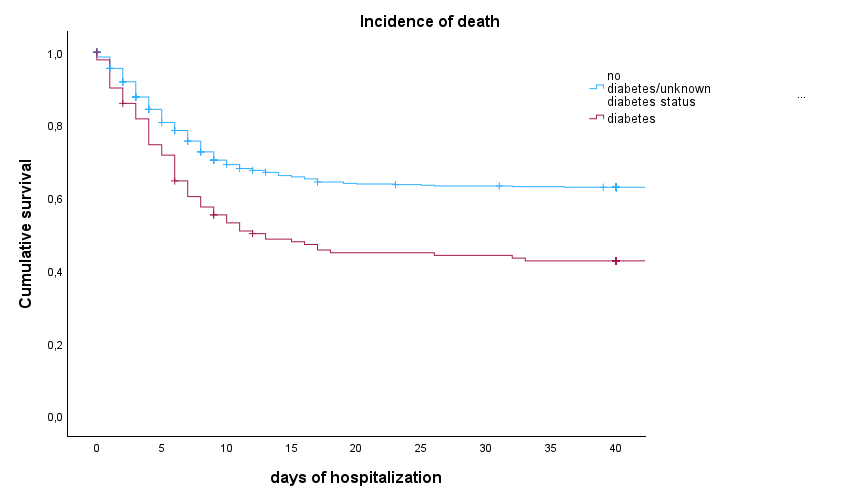
**

**B**

**
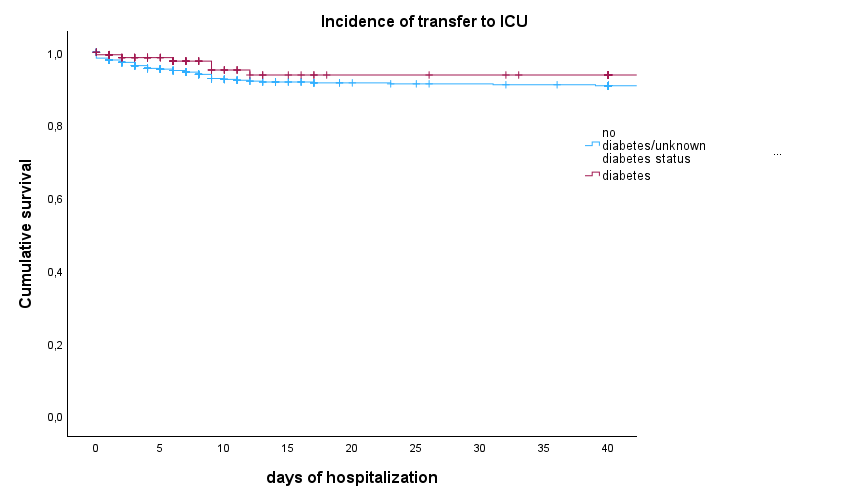
**

**Figure** **SI2.** Cumulative survival rates for composite endpoint during hospitalization according to hyperglicemia (FPG≥126 mg/dl).

**
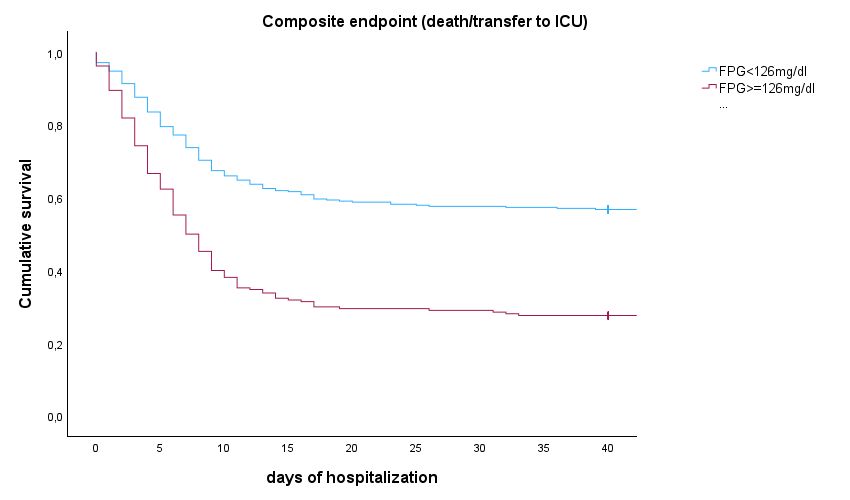
**
